# Organoid models combined with genome engineering and epigenome studies to define SOX2 function evolution in esophageal squamous cell carcinoma

**DOI:** 10.1111/1759-7714.14118

**Published:** 2021-08-29

**Authors:** Fei‐Yu Diao

**Affiliations:** ^1^ Department of General Surgery, Sun Yat‐Sen Memorial Hospital Sun Yat‐Sen University Guangzhou China

Esophageal cancer is the eighth most frequently reported malignancy and the sixth leading cause of cancer death worldwide.^1^ More than half of new esophageal cancer cases and deaths, of which about 90% are esophageal squamous cell carcinoma (ESCC), are in China.^2^, ^3^ Effective therapeutic options are limited for patients with advanced ESCC.^4^, ^5^ Cytogenetic abnormalities, such as gain of chromosome 3q which targets the locus encoding sex‐determining region Y‐box 2 (*SOX2*), is a common event in ESCC.^6^ The genomic amplification of *SOX2* has been identified as one of the most specific genomic alterations in ESCC, indicating that SOX2 plays a vital role in ESCC initiation and progression.^6^

SOX2 is a well known cell fate determining transcription factor and octamer‐binding transcription factor 4 (OCT4) protein partner active within embryonic and pluripotent stem cells. However, SOX2 plays an important role in promoting development of the esophagus and is a marker of precursor populations in the adult esophagus.^7^ In other words, SOX2 can perform distinct functions in cells of different lineages. This is because it has multiple binding sites which can interact with different protein partners. Indeed, Watanabe et al.^8^ demonstrated that SOX2 in squamous cell carcinoma could directly bind to p63 (a squamous transcription factor) and colocalize in specific genomic regions. So far, we know that both SOX2 and p63 are coexpressed in normal esophageal squamous progenitor cells and ESCC cells,^9^ but the difference between the normal and neoplastic functions of SOX2 is still unknown. Distinguishing between the two types of SOX2 function and their potential molecular mechanisms will enable better understanding of the initiation and progression of ESCC and promote the development of targeted therapy.

In a study recently published in *Nature Genetics*, “Reprogramming of the esophageal squamous carcinoma epigenome by SOX2 promotes ADAR1 dependence”, Wu et al.^10^ defined the evolution of SOX2 function during ESCC carcinogenesis by establishing a set of engineered organoids representing phenotypes from the normal esophagus to SOX2‐induced ESCC. Specifically, they compared SOX2 activity in normal esophagus and malignant tissues at different stages and characterized the epigenetic and transcriptional programs induced by SOX2 during the evolution from normal to cancer. They found that SOX2 maintained most of the functions observed in normal esophageal tissues, but when the cells in the esophagus undergo SOX2 overexpression and tumor suppressor gene (*p53* and *p16*) inactivation simultaneously, chromatin remodeling and SOX2 cistrome evolution will be promoted. This leads to the opening of the loci where SOX2 binds with its protein partners, including krueppel‐like factor 5 (Klf5), AP‐1, and TEA domain transcription factor (TEAD), followed by the activation of new gene expression programs. These findings are consistent with the data provided by Dodonova et al.^11^ (both studies have demonstrated that SOX2 has the ability to promote nucleosome remodeling).

The authors in this study subsequently focused on Klf5. The function of Klf5 in ESCC is still controversial. Some research groups have demonstrated that Klf5 could promote ESCC progression,^6^ while others found that Klf5 inhibited tumor growth in ESCC.^12^ Klf5 is routinely expressed in normal esophageal epithelial cells and suggests that it may be a tumor suppressor gene,^12^ but Klf5 amplification has frequently been found in ESCC and suggests that it may act as an oncogene.^6^ This study supports the oncogenic role of Klf5. The authors found that with the assistance of Klf5, SOX2 could acquire new genomic binding sites and enhance the transcriptional activity of certain oncogenes, such as signal transducer and activator of transcription 3 (*STAT3*). Similarly, a recent study reported that Klf5, SOX2, and p63 could jointly regulate gene expression, epigenetic modification, and chromatin accessibility in ESCC.^9^ After discovering the network of SOX2 and Klf5 binding sites activated during the malignant transformation of esophageal epithelial cells, we wondered whether the ability of SOX2 to bind to new transcription factor binding sites with Klf5 may serve a series of physiological functions in specific cellular contexts. Based on the concept of cancer as a nonhealing wound, we believe that the SOX2/Klf5 transcription complex may be activated during injury repair or acute stress, and this process is hijacked by ESCC cells. Indeed, most of the pathways activated by SOX2 in ESCC, such as the YAP/TEAD and IL6/JAK/STAT signaling pathways, are associated with injury and infection. In addition, Klf5 has been reported to be a key factor involved in colon injury.^13^

This study also found that SOX2 overexpression could induce the expression of double‐stranded RNAs (dsRNAs), endogenous retroviral elements (ERVs), and interferons (IFNs), as well as enhance the dependence on double‐stranded RNA‐specific adenosine deaminase 1 (ADAR1). Since ERVs are considered to be the markers of injury or acute stress and can enhance IFN response by stimulating dsRNA sensors, SOX2‐induced ERVs may be a byproduct of injury or acute stress. Furthermore, SOX2‐induced ERVs can promote chronic inflammatory conditions^14^ reflecting the potential role of chronic inflammation to promote carcinogenesis. It is worth noting that ERVs have also been shown to regulate the self‐renewal of stem cells.^15^ Therefore, we believe that SOX2‐induced ERVs may not only activate innate immune signaling, but also contribute to enhancing the self‐renewal of cancer stem cells. The data regarding ADAR1 dependence in this study has multiple implications. First, this study indicated that inhibiting ADAR1 may suppress ESCC growth. ADAR1 inhibition has previously been shown to enhance the efficacy of immune checkpoint inhibitors (ICIs) in cancer treatment,^16^ therefore, the combination of ADAR1 targeted therapies and ICIs may be more effective against ESCC. Moreover, the findings in this study that SOX2 promotes ERV expression in ESCC can help us determine treatment plans that enhance ICI efficacy. As epigenomic therapies can inhibit ERV expression with resulting IFN induction and ICI potentiation, combining epigenetic therapies with ADAR1 inhibitors is expected to become a highly effective cancer treatment strategy.

Overall, this study shows that combining organoid models with genome engineering and epigenome studies is an effective way to track the evolution of transcription factors involved in cancer initiation and progression. By using this strategy, the key transcription factors that drive tumorigenesis can be identified and ultimately promote the development of anticancer therapies.

## CONFLICT OF INTEREST

The author declares no competing interests.
